# The Role and Necessity of Sentinel Lymph Node Biopsy for Invasive Melanoma

**DOI:** 10.3389/fmed.2019.00231

**Published:** 2019-10-22

**Authors:** Yasuhiro Nakamura

**Affiliations:** Department of Skin Oncology/Dermatology, Saitama Medical University International Medical Center, Saitama, Japan

**Keywords:** melanoma, lymphatic metastasis, sentinel lymph node biopsy, completion lymph node dissection, observation, adjuvant therapy

## Abstract

Sentinel lymph node biopsy (SLNB) is a widely accepted procedure for melanoma staging and treatment. The development of lymphatic mapping and SLNB, which was first introduced in 1992, has enabled surgeons to detect microscopic nodal metastases and stage-negative regional nodal basins with low morbidity. SLNB has also facilitated the selective application of regional lymph node dissection for patients with microscopic nodal metastases, enabling unnecessary lymph node dissection. In contrast, recent major randomized phase III trials (DeCOG-SLT and MSLT–II trial) compared the clinical benefit of early completion lymph node dissection with observation after detecting microscopic nodal disease. The results of those studies indicated that there was no significant difference in the survival between the two groups, although regional control was superior after early completion lymph node dissection compared to that obtained after observation. Thus, the role and value of early completion lymph node dissection worldwide are currently very limited for patients with microscopic nodal disease. However, the use of SLNB is still controversial. In addition, the recent approval of adjuvant therapy using novel agents, such as anti-programmed death-1 antibodies, and molecular targeted therapeutics may influence the skipping of complete lymph node dissection in patients with micrometastatic nodal disease in a real-world setting. Furthermore, modern neoadjuvant therapy, which is now under investigation, may have the potential to change the surgical procedure used for nodal disease. Herein, we describe the current role and value of SLNB and completion lymph node dissection and discuss the major controversies as well as the favorable future outlook.

## Introduction

Malignant melanoma is among the most common types of cancer, with an increasing incidence rate of 7.9 per 100,000 people in 1975 to 25.8 per 100,000 people in 2015 (1). Approximately 7% of patients are diagnosed with stage III disease, who have a 5 year survival rate was 60.8% (2). The treatment approach for stage III patients is crucial because cutaneous melanoma often metastasizes first to the regional lymph nodes and the sentinel lymph node (SLN) is the first lymph node to receive lymphatic drainage from the primary site. The surgical approach for treating regional lymph node metastasis has continued to develop, particularly considering sentinel lymph node biopsy (SLNB). Although most patients with melanoma have no clinical nodal disease at the first visit, some patients have clinically undetectable micrometastasis in the regional lymph node. The main controversy is whether completion lymph node dissection (CLND) improves the overall or disease-specific survival of patients with SLN micrometastasis. Furthermore, the advent of promising systemic therapies, confirmed in recent clinical trials, and the results of several trials, confirming the efficacy of SLNB and immediate CLND in patients with positive SLN, may drastically change the conventional methods used for surgical control of the regional lymph nodes by using CLND for all patients with a positive SLNB.

## Application of SLNB

SLNB is the most appropriate technique for accurate staging of clinical stage I and II disease. The main risk variables associated with higher SLN metastasis are Breslow thickness (BT), ulceration, and a number of mitoses. Per the 8th edition cancer staging guidelines recommended by the American Joint Committee on Cancer (AJCC) (3), SLNB is generally not recommended for melanoma patients with a BT of <0.8 mm without ulceration because the probability of a positive SLN is <5%. However, SLNB should generally be considered for melanoma patients with clinical stage IB or II disease, with the following considerations:

1. T1b (BT of <0.8 mm with ulceration or BT of 0.8 mm^−1^ mm with or without ulceration) or T1a lesions with BT <0.8 mm with other adverse features [e.g., very high mitotic index ≤ 2/mm^2^ (particularly in young patients), lymphovascular invasion, or a combination of these factors], because the probability of a positive SLN is 5–10%. SLNB should be considered for these patients after discussion.
2. Stage IB (T2a) or II (BT of >1 mm with any feature), because the probability of a positive SLN is >10%. SLNB should be offered SLNB for these patients after discussion.

No globally accepted protocols are available for processing SLNs. However, small metastases are overlooked in conventional processing, which involves the examination of a single routine hematoxylin-eosin (HE)-stained section obtained by bivalving the SLN along the long axis (4, 5). In another procedure, the SLN is sectioned serially along the short axis (breadloaf technique) to increase the amount of subcapsular tissue in the HE-stained sections (6). When routine H&E staining does not reveal SLN metastases, immunohistochemistry (IHC) for S100, HMB-45, and MART-1/Melan-A is useful for detecting additional SLN-positive patients (7, 8). Reverse transcription-polymerase chain reaction (RT-PCR) and cell culture can increase the detection rates of positive SLN even when there are only a few metastatic melanoma cells in the SLN (9); however, these molecular biology techniques are not widely used in most institutions.

## Management of Patients With Positive SLN: Results of Recent Studies and the Role of SLNB

When patients show positive results for SLN metastasis, CLND has traditionally been indicated. However, the findings of recent studies regarding the therapeutic value of SLNB and immediate CLND after positive SLNB have resulted in a change in this traditional strategy.

## Dermatologic Cooperative Oncology Group-Sentinel Lymph node Trial (DeCOG-SLT)

The Dermatologic Cooperative Oncology Group-Sentinel Lymph node Trial (DeCOG-SLT), conducted in Germany, was the first phase III randomized clinical trial to evaluate the efficacy of immediate CLND in patients with melanoma on the trunk and limbs with BT of ≥1.0 mm and positive SLN (10). The patients were randomly assigned to the immediate CLND group (*n* = 240) or the observation group (*n* = 233; patients underwent delayed CLND only if regional metastasis was suspected on ultrasonography performed every 3 months). There were no significant differences in the distant metastasis-free survival, recurrence-free survival, and overall survival (OS) between the two groups. In this study, most patients (*n* = 311) had SLN tumor burdens of ≤1.0 mm. This high proportion of SLN micrometastasis leads to the high probability of negative non-SLN in both groups. There was no significant difference in distant metastasis-free survival between the two groups in this cohort. Therefore, distant metastasis-free survival in the cohort with SLN tumor burdens of >1.0 mm was also analyzed. There was no significant difference in the distant metastasis-free survival between the two groups, but the sample size was small in each group (*n* = 62 in the CLND group and *n* = 59 in the observation group). The authors concluded that immediate CLND was not associated with improved distant metastasis-free survival, recurrence-free survival, and OS after a median follow-up of 72 months, and no longer recommend CLND for patients with micrometastases.

## Multicenter Selective Lymphadenectomy Trial (MSLT-II)

MSLT–II enrolled a large number of patients with positive SLN (9). This was also a multicenter, phase III randomized trial that compared the immediate CLND group (*n* = 824) with the observation group (*n* = 931; patients underwent CLND only when regional metastasis was suspected on ultrasonography performed every 4 months). The mean 3 year melanoma-specific survival rate was statistically insignificant between the two groups after a median follow-up of 43 months. The disease-free survival (DFS) was slightly significantly better in the CLND group than in the observation group (*P* = 0.05). A positive non-SLN status was a reliable, independent prognostic factor for recurrence [hazard ratio (HR), 1.78; *P* = 0.005]. The occurrence of post-operative lymphedema was higher in the CLND group (24.1%) than in the observation group (6.3%). Likewise, the authors concluded that immediate CLND was not associated with improved melanoma-specific survival, but improved the regional recurrence rate and provided prognostic information.

## How are Patients Harboring Positive SLN Managed?

The above-mentioned two randomized trials demonstrated no survival benefit even if patients received immediate CLND after positive SLNB, although the nodal recurrence rate decreased in the immediate CLND group. The results of these trials do not recommend routine CLND in most patients after positive SLNB. However, their conclusions are still limited, as most patients in these studies had lower tumor burdens in the SLN (>60%). Those populations have a low probability of positive non-SLN in both groups. The true efficacy of immediate CLND after positive SLNB in patients with a higher risk, with SLN tumor burdens of >1 mm, is still unknown because of the small sample size in these trials. Therefore, current NCCN guidelines still recommend CLND, along with careful observation in patients with positive SLN after appropriate risk stratification (11). Accordingly, some guides, such as nomograms, should be utilized for accurate prediction of non-SLN status, regional control, and prognosis. This will enable us to conduct clinical trials for confirming the survival advantage of CLND in a more homogenous cohort with positive non-SLNs. Previously published predictive models for positive non-SLNs (12–18) are shown in Table 1. Although several studies have suggested similar clinicopathological characteristics as predictive parameters, a recent study by Bertolli et al. proposes BT, the number of positive SLNs, and large tumor diameter as significant predictive parameters, using their nomogram (18). This model shows the best discriminatory power (AUC 0.752) and Brier score (0.085) among all published predictive models (18) (Table 1).

**Table 1:** The performance of published prediction models for non-sentinel lymph node positivity.

**References**	**Patient no. for research**	**Significant clinicopathological parameters**	**Discrimination AUC (95% CI)**	**Calibration brier score (95% CI)**
Lee et al. (12)	191	Breslow thickness SLN tumor burden diameter	0.65 (0.60–0.70)	0.19 (0.18–0.20)
Sabel et al. (13)	221	Sex Breslow thickness Extranodal extension in SLN No. of positive SLNs	0.67 (0.63–0.74)	0.18 (0.16–0.20)
Gershenwald et al. (14)	343	SLN tumor burden diameter Breslow thickness No. of SLNs harvested	0.65 (0.60–0.70)	0.18 (0.17–0.20)
Murali et al. (15)	309	Sex Primary tumor regression No. of positive SLNs SLN tumor burden diameter SLN metastasis site	0.65 (0.60–0.70)	0.18 (0.17–0.19)
Kibrite et al. (16)	171	Breslow thickness SLN tumor burden diameter	0.65 (0.60–0.70)	0.19 (0.18–0.20)
Rossi et al. (17)	1220	Breslow thickness Primary tumor site SLN tumor burden diameter SLN metastasis site No. of SLNs harvested No. of positive SLNs	0.74 (0.70–0.79)	0.16 (0.15–0.17)
Bertolli et al. (18)	1213	Breslow thickness No. of positive SLNs SLN tumor burden diameter	0.86 (0.73–0.99)	0.085 (N.A.)

*SLN, sentinel lymph node; AUC, area under the curve; CI, confidence interval; N.A., not available*.

The racial difference in the proportion of clinical type is also crucial for considering the role of SLNB and immediate CLND. For example, acral melanoma (AM) shows drastic differences from other clinical types considering the biological, genetic, and clinicopathological aspects, although SLNB is also widely applied in clinical practice. The actual role of SLNB in this cohort remains unclear, as limited number of AM patients were included in the large trials of SLNB (i.e., DeCOG-SLT and MSLT-II) that mainly investigated Caucasian people (9, 10, 19). Ito et al. retrospectively investigated Japanese AM patients (*n* = 116) who received SLNB (20). Positive SLN was associated with significantly shorter melanoma-specific survival and DFS. The impact of positive SLNs on melanoma-specific survival was increased in AM patients with >1 mm thickness (5 year survival, 22.7 vs. 80.8%; *P* = 0.0005). Although the sample size was small in these studies, the trends of positive SLN status in association with more frequent recurrence and worsened survival in AM patients were similar to those trends in larger prospective randomized trials; however, there are no data regarding the efficacy of immediate CLND compared with observation.

## Role of Adjuvant Therapy for Positive SLN Patients Without Immediate CLND

The recent development of novel agents, including immune checkpoint inhibitors (ICIs) and molecular target agents, and their approval in many countries worldwide changed the treatment strategy for not only disease in the advanced stage but also treatment in the post-operative adjuvant setting. All these clinical trials mainly included stage III patients who underwent CLND and no patients skipped CLND after positive SLNB.

## Anti-CTLA-4 Antibodies

A phase III randomized controlled trial (EORTC 18071) comparing ipilimumab with placebo for stage III melanoma patients indicated a significant improvement in the 3 year relapse-free survival (RFS), distant metastasis-free survival, and OS in the ipilimumab group (21). However, severe immune-related adverse events were observed in 41.6% of patients in the ipilimumab group, leading to discontinuation of ipilimumab in half of the patients.

## Anti-PD-1 Antibodies

The clinical benefits of two anti-PD-1 agents as adjuvant therapy were reported recently. A phase III randomized controlled trial (Checkmate 238) comparing nivolumab with ipilimumab for stage IIIB to IV melanoma patients (22) demonstrated better 1 year RFS with lower toxicity in the nivolumab group than in the ipilimumab group. Likewise, a phase III randomized trial (KEYNOTE-054) comparing pembrolizumab with placebo for stage III patients, except for <1 mm of tumor burden in the SLN, also demonstrated improvement in the recurrence-free survival of patients receiving pembrolizumab compared to those receiving placebo after a median follow-up of 15 months (HR, 0.57; *P* < 0.001) (23).

## BRAF Inhibitor/MEK Inhibitor

A phase III randomized trial (COMBI-AD) comparing dabrafenib plus trametinib with placebo for patients with stage III BRAF mutant melanoma, except for <1 mm of tumor burdens in the SLN, showed improved RFS in the dabrafenib/trametinib group after a median follow-up of 44 months in the dabrafenib/trametinib group and 42 months in the placebo group (24). There also was a trend of improvement in the OS [the 3-year OS rate was 86% in the dabrafenib/trametinib group and 77% in the placebo group (HR, 0.57; *P* = 0.0006)], although the data obtained on statistical analysis did not fulfill the pre-specified interim analysis boundary (*P* = 0.000019) (25).

Based on the above-mentioned clinical trials, the latest NCCN guidelines recommend adjuvant nivolumab for stage IIIB/C and IV melanoma patients after complete tumor removal. Pembrolizumab was recommended for stage III melanoma patients with ≥1 mm tumor burden in the SLN. In patients with BRAF mutations, dabrafenib plus trametinib can also be alternatively recommended for stage III disease with ≥1 mm tumor burden in the SLN.

The result of SLNB can be used to classify patients without clinical nodal disease for undergoing adjuvant therapy. However, all the above-mentioned clinical trials required CLND before initiating adjuvant therapy. Conversely, in the real-world setting, patients who have positive SLN and do not undergo CLND will increase considering the results of the DeCOG-SLT and MSLT-II trials, even if the patients' tumor burdens exceed 1 mm. Currently, there are no data about the survival benefit of adjuvant therapy with the novel agents in patients who skipped CLND after positive SLNB. Therefore, further research is required to investigate the survival differences between the clinical trial populations and the more heterogeneous real-world population.

## Possible Role of Neoadjuvant Therapy

The reports of modern neoadjuvant clinical trials using ICIs or molecular targeted agents demonstrate promising efficacy, mainly for clinical stage III disease. All agents for neoadjuvant use have not yet been approved worldwide.

## Anti-PD-1 Antibodies and Anti-PD-1/Anti-CTLA-4 Antibody

Huang et al. conducted a phase Ib trial investigating the safety of neoadjuvant/adjuvant pembrolizumab for resectable clinical stage III and IV melanoma (26). Enrolled patients received neoadjuvant/adjuvant pembrolizumab (1 cycle of neoadjuvant pembrolizumab 3 weeks before surgery and 17 cycles of adjuvant pembrolizumab). Eight of 27 patients (30%) achieved complete or major pathological response, and they remain free of disease.

Amaria et al. reported a randomized phase II trial comparing the efficacy and safety of neoadjuvant nivolumab (four cycles of neoadjuvant and 13 cycles of adjuvant nivolumab) to neoadjuvant nivolumab/ipilimumab (three cycles of neoadjuvant nivolumab/ipilimumab and 13 cycles of adjuvant nivolumab) for resectable clinical stage III and IV melanoma (27). Neoadjuvant nivolumab/ipilimumab demonstrated higher response rates (RRs) [objective RR, 73 vs. 25%; pathological complete response (pCR), 45 vs. 25%] but also showed higher toxicity (grade 3 treatment-related adverse events, 73 vs. 8%).

Blank et al. also reported a randomized phase II trial (OpACIN) comparing neoadjuvant nivolumab/ipilimumab (two cycles of neoadjuvant nivolumab/ipilimumab and two cycles of adjuvant nivolumab/ipilimumab) with adjuvant nivolumab/ipilimumab (four cycles of adjuvant nivolumab/ipilimumab) in patients with palpable stage III melanoma (28). The neoadjuvant arm achieved high pathological responses (78%), and no patients showing response developed recurrence during the median follow-up or 25.6 months. However, 9 of 10 patients experienced grade 3/4 adverse events in both treatment arms.

Rozeman et al. conducted a phase II randomized trial (OpACIN-neo) comparing three different doses and cycles of neoadjuvant nivolumab/ipilimumab for resectable clinical stage III melanoma (29). The following were three protocols: group A, two cycles of nivolumab (1 mg/kg) and ipilimumab (3 mg/kg); group B, two cycles of nivolumab (3 mg/kg) and ipilimumab (1 mg/kg); and group C, two cycles of ipilimumab (3 mg/kg) followed by two cycles of nivolumab (3 mg/kg). The objective radiological and pathological RRs were 63% (19/30) and 80% (24/30) in group A, 57% (17/30) and 77% (23/30) in group B, and 35% (9/26) and 65% (17/26) in group C, respectively. The rate of grade 3/4 immune-related adverse events was lower in group B than in groups A and C (group A, 40% [12/30]; group B, 20% [6/30]; group C, 50% [13/26]). One group A patient died of encephalitis.

## BRAF Inhibitor/MEK Inhibitor

Amaria et al. reported a randomized phase II trial for patients with resectable clinical stage III or oligometastatic stage IV melanoma harboring BRAFV600E/K mutation (30). The patients were randomly assigned to either undergo surgery followed by adjuvant therapy without ICIs or targeted agents or to receive neoadjuvant/adjuvant dabrafenib/trametinib (8 weeks of neoadjuvant and 44 weeks of adjuvant dabrafenib/trametinib). The neoadjuvant group showed significantly long event-free survival (median event-free survival: 19.7 vs. 2.9 months; HR 0.016; *P* < 0.0001).

Long et al. also reported a single-arm phase II trial (NeoCombi) for patients with resectable clinical stage IIIB-C (AJCC 7th edition) melanoma harboring BRAFV600 mutation (31). The patients received neoadjuvant/adjuvant dabrafenib/trametinib (12 weeks of neoadjuvant and 40 weeks of adjuvant dabrafenib/trametinib). Thirty of 35 patients (86%) achieved a response (46%, complete response; 40%, partial response). All patients achieved pathological response, including 17 patients (49%) with pCR.

These novel neoadjuvant therapies, involving active regimens mainly for clinical stage III melanoma, showed high pathological RRs. Remarkably, no patients achieving pCR after treatment with ICIs developed recurrence during the follow-up periods. However, these esults must be interpreted with caution as these trials did not report OS after long-termfollow-up.

## Immunology of SLNs

Immunohistological and molecular characteristics of SLNs may be useful in predicting the development of regional or distant metastasis, because the SLN represents the immunological site at which anti-tumor immune dysfunction is established and where potential prognostic immunologic markers can be found. Considering the immunologic microenvironment, the number of CD3+, CD4+, and CD8+ tumor-infiltrating lymphocytes in positive SLN is associated with better recurrence-free survival and OS (32). Elevated levels of regulatory T cell markers, such as FOXP3 and indoleamine 2,3-dioxygenase, correlate with increasing rates of local, regional, and distant metastases (33, 34). A study focused on regression of the primary tumor indicated that a regression of more than 10% was a reliable cutoff to divide different risk categories (35). Only a small number of CD4+/CD25+, FOXP3+/CD4+, or PD1+/CD4+ lymphocytes infiltrated the regressed areas. These lymphocytes were correlated with anergy and lower CD8+ lymphocyte immune response to melanoma cells. Thus, these findings may help in developing novel therapeutic strategies for selecting SLNB and immediate CLND for patients with stage III melanoma. As for molecular characteristics, Vallacchi et al. reported a pilot study involving integrated analysis of genome-wide transcriptional profiles and *in vitro* assessment of immune cells present in positive SLNs. This analysis identified microRNA, involved in the regulation of the TNF receptor superfamily member 8 gene that encodes the CD30 receptor, as a marker in the lymphocytes of melanoma patients with progressive disease. These findings demonstrate that microRNA is associated with the regulation of immune dysfunction in SLNs, providing a valuable prognostic molecular marker for identifying stage III melanoma patients at risk of recurrence.

## Conclusions

SLNB has contributed to the selection of earlier CLND in patients without nodal disease by detecting microscopic positive SLN. Conversely, it is questionable whether CLND is required if SLN itself was therapeutic in patients with microscopic positive SLN alone. The results of two recent randomized clinical trials suggested that immediate CLND for positive SLN patients was not associated with DFS, OS, and metastasis-free survival, despite an increased risk of delayed non-SLN recurrence. Currently, SLNB provides prognostic information and has a therapeutic role in patients with a low tumor burden with intermediate-thickness melanoma. SLNB is also useful to select patients with the appropriate stage for undergoing post-operative adjuvant therapy. Immediate CLND is no longer routinely recommended for all patients with positive SLNB, particularly for patients without suspected non-SLN metastasis. At present, immediate CLND is ideal for patients at low risk of distant metastasis but at high risk of delayed regional metastasis.

The future of SLNB and CLND will depend on the development of promising neoadjuvant/adjuvant therapies and excellent biomarkers, which may drastically change the treatment strategies for stage III melanoma patients as well as the current TNM classification. This may lead to the advent of a new era in which surgical procedures would not be required for high-risk patients, including those with stage III disease.

## Author Contributions

YN had full access to all of the data in the study and take responsibility for study concept and design, acquisition, analysis, interpretation of data, drafting of the manuscript, and critical revision of the manuscript for important intellectual content.

### Conflict of Interest

The author declares that the research was conducted in the absence of any commercial or financial relationships that could be construed as a potential conflict of interest.

## References

[1] Siegel RL Miller KD Jemal A Cancer statistics, 2018. CA Cancer J Clin. (2018) 68:7–30. 10.3322/caac.21442 29313949

[2] Cronin KA Lake AJ Scott S Sherman RL Noone AM Howlader N. Annual report to the nation on the status of cancer, part I: National Cancer Statistics. Cancer. (2018) 124:2785–800. 10.1002/cncr.31551 29786848 PMC6033186

[3] Gershenwald JE Scolyer RA Hess KR Sondak VK Long GV Ross MI. Melanoma staging: evidence-based changes in the American Joint Committee on Cancer eighth edition cancer staging manual. CA Cancer J Clin. (2017) 67:472–92. 10.3322/caac.21409 29028110 PMC5978683

[4] Prieto VG. Use of frozen sections in the examination of sentinel lymph nodes in patients with melanoma. Semin Diagn Pathol. (2008) 25:112–5. 10.1053/j.semdp.2008.04.001 18697714

[5] Gershenwald JE Colome MI Lee JE Mansfield PF Tseng C Lee JJ Patterns of recurrence following a negative sentinel lymph node biopsy in 243 patients with stage I or II melanoma. J Clin Oncol. (1998) 16:2253–60. 10.1200/JCO.1998.16.6.2253 9626228

[6] Prieto VG Clark SH. Processing of sentinel lymph nodes for detection of metastatic melanoma. Ann Diagn Pathol. (2002) 6:257–64. 10.1053/adpa.2002.35400 12170459

[7] Abrahamsen HN Hamilton-Dutoit SJ Larsen J Steiniche T. Sentinel lymph nodes in malignant melanoma: extended histopathologic evaluation improves diagnostic precision. Cancer. (2004) 100:1683–91. 10.1002/cncr.20179 15073857

[8] Yu LL Flotte TJ Tanabe KK Gadd MA Cosimi AB Sober AJ. Detection of microscopic melanoma metastases in sentinel lymph nodes. Cancer. (1999) 86:617–27. 10.1002/(SICI)1097-0142(19990815)86:4<617::AID-CNCR10>3.3.CO;2-J 10440689

[9] Faries MB Thompson JF Cochran AJ Aracena CJ Lotti T Completion Dissection or Observation for Sentinel-Node Metastasis in Melanoma. N Engl J Med. (2017) 376:2211–22. 10.1111/dth.12544 28591523 PMC5548388

[10] Leiter UM Stadler R Mauch C Hohenberger W Brockmeyer N Berking C Final analysis of DECOG-SLT trial: survival outcomes of complete lymph node dissection in melanoma patients with positive sentinel node. J Clin Oncol. 36(15_supp):9501 10.1200/JCO.2018.36.15_suppl.9501 31557067

[11] Fiddian-Green RG Silen W NCCN Clinical Practice Guidelines in Oncology (NCCN Guidelines^?^) Melanoma Version 2.2019. (2019). Available online at: https://www.nccn.org/professionals/physician_gls/pdf/cutaneous_melanoma.pdf (accessed March 12, 2019).

[12] Lee JH Essner R Torisu-Itakura H Wanek L Wang H Morton DL. Factors predictive of tumor-positive nonsentinel lymph nodes after tumor-positive sentinel lymph node dissection for melanoma. J Clin Oncol. (2004) 22:3677–84. 10.1200/JCO.2004.01.012 15365064

[13] Sabel MS Griffith K Sondak VK Lowe L Schwartz JL Cimmino VM. Predictors of nonsentinel lymph node positivity in patients with a positive sentinel node for melanoma. J Am Coll Surg. (2005) 201:37–47. 10.1016/j.jamcollsurg.2005.03.029 15978442

[14] Gershenwald JE Andtbacka RH Prieto VG Johnson MM Diwan AH Lee JE. Microscopic tumor burden in sentinel lymph nodes predicts synchronous nonsentinel lymph node involvement in patients with melanoma. J Clin Oncol. (2008) 26:4296–303. 10.1200/JCO.2007.15.4179 18606982 PMC2653121

[15] Murali R Desilva C Thompson JF Scolyer RA. Non-Sentinel Node Risk Score (N-SNORE): a scoring system for accurately stratifying risk of non-sentinel node positivity in patients with cutaneous melanoma with positive sentinel lymph nodes. J Clin Oncol. (2010) 28:4441–9. 10.1200/JCO.2010.30.9567 20823419

[16] Kibrité A Milot H Douville P Gagné ÉJ Labonté S Friede J. Predictive factors for sentinel lymph nodes and non-sentinel lymph nodes metastatic involvement: a database study of 1,041 melanoma patients. Am J Surg. (2016) 211:89–94. 10.1016/j.amjsurg.2015.05.016 26275921

[17] Rossi CR Mocellin S Campana LG Borgognoni L Sestini S Giudice G. Prediction of non-sentinel node status in patients with melanoma and positive sentinel node biopsy: an Italian Melanoma Intergroup (IMI) study. Ann Surg Oncol. (2018) 25:271–9. 10.1245/s10434-017-6143-5 29067603

[18] Bertolli E Franke V Calsavara VF de Macedo MP Pinto CAL van Houdt WJ Validation of a nomogram for non-sentinel node positivity in melanoma patients, and its clinical implications: a Brazilian-Dutch study. Ann Surg Oncol. (2019) 26:395–405. 10.1245/s10434-018-7038-9 30456681

[19] Morton DL Thompson JF Cochran AJ Mozzillo N Nieweg OE Roses DF. Final trial report of sentinel-node biopsy versus nodal observation in melanoma. N Engl J Med. (2014) 370:599–609. 10.1056/NEJMoa1310460 24521106 PMC4058881

[20] Ito T Wada M Nagae K Nakano-Nakamura M Nakahara T Hagihara A. Acral lentiginous melanoma: who benefits from sentinel lymph node biopsy? J Am Acad Dermatol. (2015) 72:71–7. 10.1016/j.jaad.2014.10.008 25455840

[21] Eggermont AM Chiarion-Sileni V Grob JJ Dummer R Wolchok JD Schmidt H. Prolonged survival in stage III melanoma with ipilimumab adjuvant therapy. N Engl J Med. (2016) 375:1845–55. 10.1056/NEJMoa1611299 27717298 PMC5648545

[22] Weber J Mandala M Del Vecchio M Gogas HJ Arance AM Cowey CL Adjuvant nivolumab versus ipilimumab in resected stage III or IV melanoma. N Engl J Med. (2017) 377:1824–35. 10.1056/NEJMoa1709030 28891423

[23] Eggermont AMM Blank CU Mandala M Long GV Atkinson V Dalle S. Adjuvant pembrolizumab versus placebo in resected stage III melanoma. N Engl J Med. (2018) 378:1789–801. 10.1056/NEJMoa1802357 29658430

[24] Hauschild A Dummer R Schadendorf D Santinami M Atkinson V Mandalà M Longer follow-up confirms relapse-free survival benefit with adjuvant dabrafenib plus trametinib in patients with resected BRAF V600-mutant stage III melanoma. J Clin Oncol. (2018) 2018:JCO1801219 10.1200/JCO.18.01219 PMC6286159 30343620

[25] Long GV Hauschild A Santinami M Atkinson V Mandalà M Chiarion-Sileni V. Adjuvant dabrafenib plus trametinib in stage III BRAF-mutated melanoma. N Engl J Med. (2017) 377:1813–23. 10.1056/NEJMoa1708539 28891408

[26] Huang AC Orlowski RJ Xu X Mick R George SM Yan PK. A single dose of neoadjuvant PD-1 blockade predicts clinical outcomes in resectable melanoma. Nat Med. (2019) 25:454–61. 10.1038/s41591-019-0357-y 30804515 PMC6699626

[27] Amaria RN Reddy SM Tawbi HA Davies MA Ross MI Glitza IC Neoadjuvant immune checkpoint blockade in high-risk resectable melanoma. Nat Med. (2018) 24:1649–54. 10.1038/s41591-018-0197-1 30297909 PMC6481682

[28] Blank CU Rozeman EA Fanchi LF Sikorska K van de Wiel B Kvistborg P. Neoadjuvant versus adjuvant ipilimumab plus nivolumab in macroscopic stage III melanoma. Nat Med. (2018) 24:1655–61. 10.1038/s41591-018-0198-0 30297911

[29] Rozeman EA Menzies AM van Akkooi ACJ Adhikari C Bierman C van de Wiel BA. Identification of the optimal combination dosing schedule of neoadjuvant ipilimumab plus nivolumab in macroscopic stage III melanoma (OpACIN-neo): a multicentre, phase 2, randomised, controlled trial. Lancet Oncol. (2019) 20:948–60. 10.1016/S1470-2045(19)30151-2 31160251

[30] Amaria RN Prieto PA Tetzlaff MT Reuben A Andrews MC Ross MI. Neoadjuvant plus adjuvant dabrafenib and trametinib versus standard of care in patients with high-risk, surgically resectable melanoma: a single-centre, open-label, randomised, phase 2 trial. Lancet Oncol. (2018) 19:181–93. 10.1016/S1470-2045(18)30015-9 29361468

[31] Long GV Saw RPM Lo S Nieweg OE Shannon KF Gonzalez M Neoadjuvant dabrafenib combined with trametinib for resectable, stage IIIB-C, BRAF(V600) mutation-positive melanoma (NeoCombi): a single-arm, open-label, single-centre, phase 2 trial. Lancet Oncol. (2019) 20:961–71. 10.1016/S1470-2045(19)30331-6 31171444

[32] Kakavand H Vilain RE Wilmott JS Burke H Yearley JH Thompson JF. Tumor PD-L1 expression, immune cell correlates and PD-1+ lymphocytes in sentinel lymph node melanoma metastases. Mod Pathol. (2015) 28:1535–44. 10.1038/modpathol.2015.110 26403784

[33] Speeckaert R Vermaelen K van Geel N Autier P Lambert J Haspeslagh M. Indoleamine 2,3-dioxygenase, a new prognostic marker in sentinel lymph nodes of melanoma patients. Eur J Cancer. (2012) 48:2004–11. 10.1016/j.ejca.2011.09.007 22033321

[34] Ryan M Crow J Kahmke R Fisher SR Su Z Lee WT. FoxP3 and indoleamine 2,3-dioxygenase immunoreactivity in sentinel nodes from melanoma patients. Am J Otolaryngol. (2014) 35:689–94. 10.1016/j.amjoto.2014.08.009 25212103

[35] Osella-Abate S Conti L Annaratone L Senetta R Bertero L Licciardello M. Phenotypic characterisation of immune cells associated with histological regression in cutaneous melanoma. Pathology. 2019. 10.1016/j.pathol.2019.04.001 31266597

